# Safety, Tolerability, Pharmacokinetics, and Concentration-QTc Analysis of Tetrodotoxin: A Randomized, Dose Escalation Study in Healthy Adults

**DOI:** 10.3390/toxins12080511

**Published:** 2020-08-09

**Authors:** Mojgan Kavoosi, Terry E. O’Reilly, Mehran Kavoosi, Peng Chai, Caroline Engel, Walter Korz, Christopher C. Gallen, Robert M. Lester

**Affiliations:** 1 WEX Pharmaceuticals Inc., Vancouver, BC V6E-4A6, Canada; mehrank@wexpharma.com (M.K.); walterk@wexpharma.com (W.K.); christopherg@wexpharma.com (C.C.G.); 2 Celerion Inc., Tempe, AZ 85283, USA; terry.oreilly@celerion.com (T.E.O.); robert.lester@celerion.com (R.M.L.); 3 Celerion Inc., Lincoln, NE 68502, USA; peng.chai@celerion.com; 4 Celerion Inc., Montreal, QC H4M-2P1, Canada; caroline.engel@celerion.com

**Keywords:** tetrodotoxin, pain, analgesic, QT interval, ECG, voltage-gated sodium channels, clinical trial, neuropathy, toxicity, safety

## Abstract

Tetrodotoxin (TTX) is a highly specific voltage-gated sodium channel (VGSC) blocker in clinical evaluation as a peripheral-acting analgesic for chronic pain. This study presents the first published results of the safety including cardiac liability of TTX at therapeutic-relevant concentrations in twenty-five healthy adults. Randomized, double-blind, placebo-, and positive- (moxifloxacin) controlled study evaluated single ascending doses of 15 µg, 30 µg, and 45 µg TTX over 3 periods with a 7-day washout between each period. Subcutaneous injections of TTX were readily absorbed, reaching maximum plasma concentration (C_max_) within 1.5 h. Both extent of exposure (AUC) and C_max_ increased in proportion to dose. No QT prolongation was identified by concentration-QTc analysis and the upper bounds of the two-sided 90% confidence interval of predicted maximum baseline and placebo corrected QTcF (ΔΔQTcF) value did not exceed 10 ms for all tetrodotoxin doses, thereby meeting the criteria of a negative QT study. Safety assessments showed no clinically relevant changes with values similar between all groups and no subject withdrawing due to adverse events. Paresthesia, oral-paresthesia, headache, dizziness, nausea, and myalgia were the most common TEAEs (overall occurrence ≥5%) in the TTX treatment groups. TTX doses investigated in this study are safe, well-tolerated, and lack proarrhythmic proclivity.

## 1. Introduction

Chronic pain is a global health problem that affects an estimated 20.4% of adults worldwide with an addition 10% of newly diagnosed cases added each year [1]. Voltage-gated sodium channels (VGSCs) are large transmembrane proteins responsible for the initiation and propagation of action potential in neurons and other excitable cells. The role of neuronal VGSCs in pain is well-established [2,3,4] and non-selective VGSC blockers namely certain anticonvulsants, antidepressants, antiarrhythmics, and local anesthetics like lacosamide, carbamazepine, lidocaine, and mexiletine, have been shown to inhibit the ectopic discharges associated with pain [5,6]. However, these compounds can often have undesirable side effects, due most likely to their non-specific activity on more than one target channel or receptor. Thus, the currently available medications for chronic pain are suboptimal. 

Tetrodotoxin (TTX) is a natural toxin used by some marine and terrestrial animals including pufferfish for defense or predation [7,8,9]. TTX binds exclusively to VGSCs and as a result, it is the gold standard used by researchers for decades to characterize the structure and function of VGSCs including distinguishing between TTX-sensitive (TTX-IC_50_ ~10 nM) and TTX-resistant (TTX-IC_50_ ≥ 1 µM) VGSCs [10,11]. TTX has important attributes as a therapeutic including high specificity for VGSCs, 1000-fold concentration differential in selectively blocking TTX-sensitive versus TTX-resistant VGSC isoforms, and inability to cross the blood-brain-barrier [12]; thus, TTX is currently undergoing evaluation as a non-addictive analgesic for chronic pain. If successful, TTX will be one of a select few peripherally acting analgesics. To date, preclinical studies have shown TTX to be effective at inhibiting neuropathic [13,14] and inflammatory pain [15,16,17]. In patients with moderate to severe pain due to either cancer or chemotherapy-induced neuropathy who failed other treatments, TTX was seen to reduce pain by 30% below baseline levels in some patients [18,19,20,21]. In addition, given the continued discovery of the role of aberrant VGSC activity in other pathophysiological conditions including cancer, migraine, and several muscle and immune system disorders [22], the therapeutic potential for TTX in these other disease conditions is significant.

Toxins, including morphine, botulinum toxin, and curare have made significant contributions in the field of pain management and their success continues to prompt the development of further analogues. Yet, despite their success and FDA approval for a TTX phase 3 clinical trial, there continue to be concerns about the safety of TTX. This safety concern includes the potential for TTX to impair the neuromuscular or respiratory system functions or induce cardiac arrythmias at therapeutic concentrations due to the prominent role of TTX-sensitive VGSCs in these systems [11,23,24] and the discovery of a low fraction of TTX-sensitive VGSCs [25,26] alongside the dominant TTX-resistant cardiac VGSC [27] on human cardiomyocytes. 

This phase 1, dose escalation study was undertaken to present the safety, tolerability, and pharmacokinetics (PK) of TTX at clinically relevant exposures. A number of respiratory and neuromuscular tests including assessment of sensory and motor system functions were performed as part of the overall safety evaluation. Finally, a concentration-QT (C-QTc) assessment was also conducted in accordance with the International Conference on Harmonization E14 guidance (ICH E14) [28] to determine the QT liability and proarrhythmic potential of TTX at therapeutically relevant concentrations.

## 2. Results

### 2.1. Subject Demographics

Twenty-five healthy subjects with a mean age of 36.7 years (range 18–53) and mean body mass index of 28.9 kg m^−2^ (range 21.4–32.0) participated in this study. The ethnic profile and baseline characteristics of participants are shown in Table 1. Of the 25 subjects enrolled, 24 completed the study. One subject in control arm (treatment sequence: moxifloxacin-placebo-moxifloxacin) withdrew due to personal reasons prior to final dosing (treatment period 3) and their data was included in the moxifloxacin PK analysis. Baseline characteristics of the overall population in the treatment and control arms were similar.

### 2.2. Dose Selection

In this study, a single dose regimen was selected after review of prior TTX pharmacokinetic analysis revealed a lack of any significant accumulation following multiple daily dose administration, and TTX was almost exclusively eliminated (approximately 96.75% of dose) via urinary excretion over 36 h as the parent compound (WEX Pharmaceuticals Inc., data on file).

TTX concentrations were selected to cover the current therapeutic range and represented a 3-fold increase in dosing. In addition, administration of 45 µg TTX was the highest dose evaluated in human trials to date and determined to be safe. The maximum plasma concentration measured after a single dose of 45 µg TTX has been determined to be greater than that of 30 µg TTX given twice a day, the commonly administered therapeutic dosage (WEX Pharmaceuticals Inc., data on file).

### 2.3. Safety and Tolerability

All the treatment emergent adverse events (TEAEs) experienced by subjects in the TTX treatment arm are shown in Table 2. There were no serious or severe AEs and no withdrawals due to AEs in this study. A total of 79 TEAEs were experienced by 8 (89%) of subjects in TTX treatment arm. Incidence of TEAEs was higher with increasing TTX doses. Mild paresthesia, a known property of TTX, was the most frequently reported AE experienced a total of 18 times by 7 (78%) subjects with onset ranging from 9 min to 2.9 days. All paresthesia events were mild and resolved without treatment. One subject developed mild tachycardia (seated pulse of 116 bpm) 42 min following administration of 15 µg TTX which resolved spontaneously without treatment approximately 2 h and 22 min post-dosing. The subject experienced no further episodes of tachycardia in period 2 (30 µg TTX) or period 3 (45 µg TTX) of the study. 

All vital signs including blood pressure (Table S1) were within normal limits for all subjects and no clinically relevant trends or significant abnormalities were observed in serum chemistry, hematology, or urinalysis values among subjects at any time during the study. No AEs based on neurological findings were observed and assessments of sensory and motor systems, including vibration, hand grip strength (Table S2), leg raises against resistance, and finger-nose coordination were unchanged and similar between all groups. Assessment of respiratory system function with pulse oximetry and peak expiratory flow test (Table S3) showed similar values between all groups at all time points evaluated. The lack of clinically observable impairment indicates therapeutic concentrations of up to 45 µg TTX do not affect motor control, muscle strength, breathing, or hinder the function of cutaneous afferent fibers responsible for detection of vibration.

### 2.4. Pharmacokinetics

**Figure 1:**
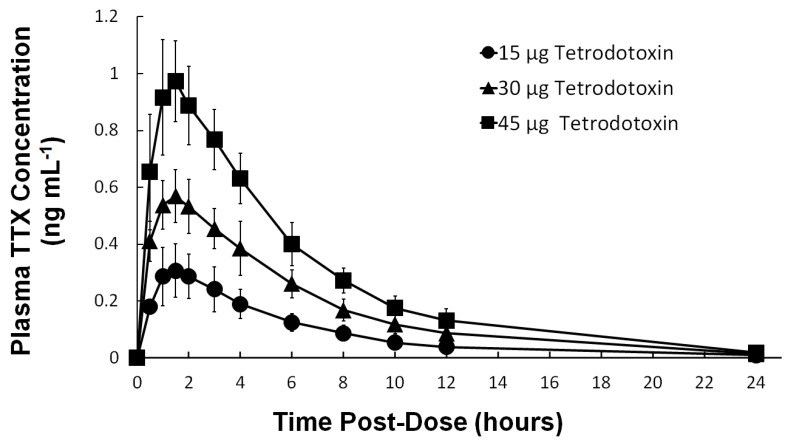
Mean (±STD) plasma TTX concentration–time profiles following single doses of 15 µg TTX (●), 30 µg TTX (▲), and 45 µg TTX (■) over three periods in this dose escalation Phase 1 study.

Plasma TTX concentration–time profile following single subcutaneous administration of 15 µg, 30 µg, and 45 µg TTX under fasting conditions is shown in Figure 1 and the corresponding plasma PK parameters are shown in Table 3. The plasma TTX concentrations increased in the first 1.5 h following dosing, reaching a maximum geometric mean concentration (geometric CV%) of 0.3046 ng mL^−1^ (30.3 CV%), 0.5807 ng mL^−1^ (17.4 CV%), and 0.9914 ng mL^−1^ (16.2 CV%) for 15 µg, 30 µg, and 45 µg TTX, respectively, which corresponds well with dosing increments. Using the maximum geometric mean concentrations, the 15 µg, 30 µg, and 45 µg TTX administered corresponds to C_max_ of approximately 0.95 nM, 1.82 nM, and 3.11 nM TTX, respectively, values below the IC_50_ for TTX-sensitive VGSCs (IC_50_ ~10 nM). Absorption and elimination of TTX were comparable across all TTX treatments as shown by similar t_½_ and t_max_ values between treatments. Plasma TTX concentration decreased steadily following t_max_ and was undetectable (below the limit of quantification) in almost all subjects at 24 h post-dose. As with C_max_, the extent of TTX exposure (AUC_0-t_ and AUC_0–∞_) increased in proportion to increasing doses. On a TTX concentration-PK parameter plot for C_max_, AUC_0–t_, and AUC_0–∞_, a slope close to one was observed (95% CI for slope includes the value of 1, graph not shown) indicating the systemic TTX concentration follows a dose proportional relationship.

### 2.5. QT Interval Correction

Fridericia’s method was used as the primary QT interval correction method $\left(QTcF=\left(QT\right)RR^{1/3}\right)$. The QTcF versus RR graphs for all treatments including moxifloxacin and placebo showed overall slope values close to zero with values of 0.0545 (95% CI: 0.0439–0.0651), 0.0477 (95% CI: 0.0303–0.0591), and 0.0367 (95% CI: 0.0234–0.0500) for TTX, moxifloxacin, and placebo, respectively. The change from baseline in heart rate values did not increase by >9 bpm in all treatment groups compared to placebo, indicating that QTcF adequately corrected for changes in heart rate and was considered appropriate for the cardiodynamic analysis in this study. 

### 2.6. QT/QTc Analysis

**Figure 2:**
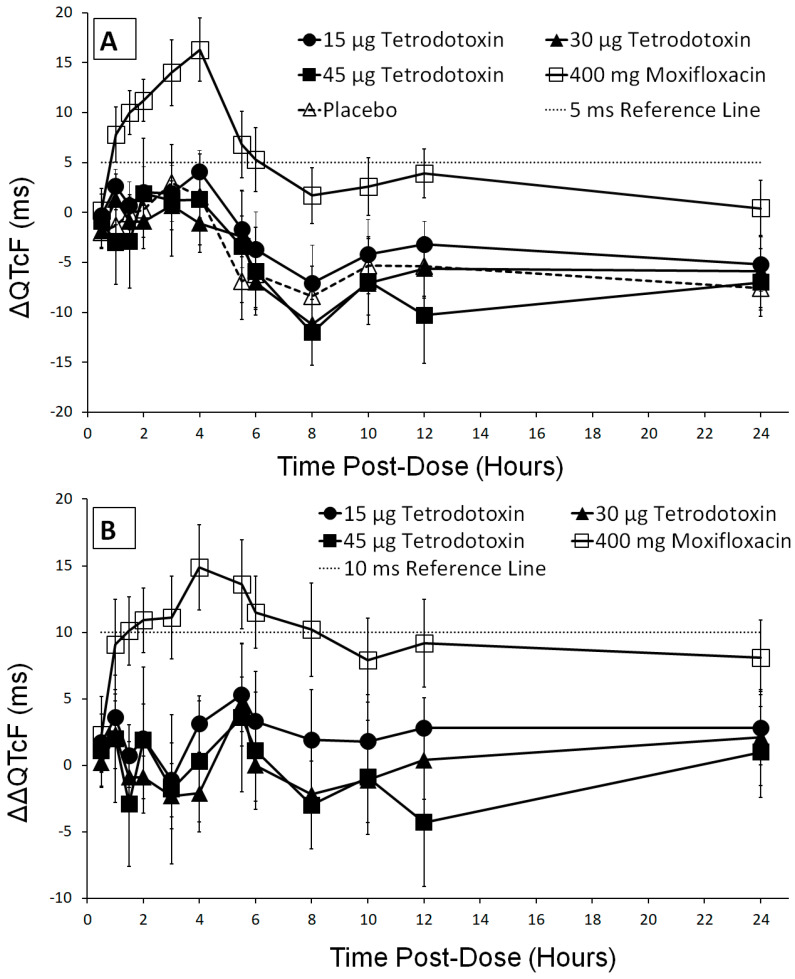
(**A**) Change from baseline in Fridericia-corrected QTc (ΔQTcF, mean ± 90% CI) and (**B**) placebo and baseline corrected QTcF (ΔΔQTcF, mean ±90% CI) in milliseconds across treatments (15 µg TTX (●), 30 µg TTX (▲),45 µg TTX (■), 400 mg moxifloxacin (□), and placebo (∆)) for all post-dose time points.

After confirming that no hysteresis was present, the effect of 15 µg, 30 µg, and 45 µg TTX doses on QT prolongation was evaluated by measuring the difference in baseline corrected QTcF values between TTX treatment and placebo groups (ΔΔQTcF). Baseline ECG parameters were comparable with average values calculated as the mean value of 9 time points prior to dosing, determined independently for each period. Maximum mean QTcF value was 406.0 ms (±25.9) observed at 2 h post-dose following administration of 45 µg TTX. One subject manifested a QT interval > 480 ms with 45 µg TTX but this did not represent an increase from baseline ≥ 60 ms. The pattern and time course of the baseline corrected QTcF (ΔQTcF) for TTX was similar to placebo with all mean values being negative or close to zero (Figure 2A). The placebo and baseline corrected QTcF (ΔΔQTcF) for all three TTX treatments showed mean values < 5 ms at all time points except for 15 µg TTX which reached a mean value of 5.3 ms at 5.5 h post-dose (Figure 2B). However, at 5.5 h post-dose, a decrease in ΔΔQTcF values with increasing TTX doses was also observed with ΔΔQTcF values of 5.3 ms (90% CI: 1.5–9.2), 4.6 ms (90% CI: 2.6–6.7), and 3.6 ms (90% CI: −2.0–9.2) for 15 µg, 30 µg, and 45 µg TTX, respectively. Upper limits of the 90% CI for all three TTX doses at all time points were below the pre-defined ICH E14 [28] threshold of 10 ms indicating that administration of doses up to 45 µg TTX does not prolong the QTcF interval. The fact that the effect on moxifloxacin was clearly greater with a peak value of 14.9 ms and mean ΔΔQTcF value > 5 ms at all post-dose time points after 0.5 h mitigates against any false negative TTX effect.

### 2.7. Concentration-QT Modeling

**Figure 3:**
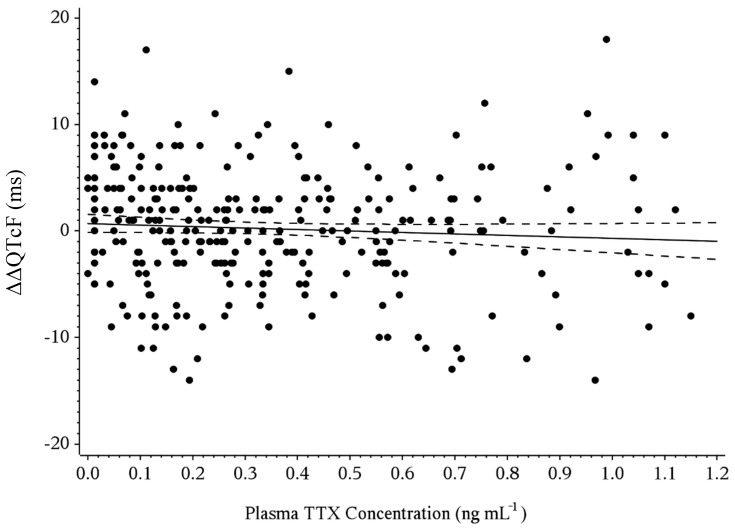
Relationship between placebo-adjusted change from baseline in QTcF (ΔΔQTcF) and tetrodotoxin plasma concentration. Slope (90% CI) = −1.378 (−3.266–0.510), *p* = 0.2294 is derived from the concentration-QT model.

Based on linear regression of ΔΔQTcF versus time-matched plasma TTX concentrations, the predicted ΔΔQTcF maximum values calculated at geometric mean C_max_ were less than 1 ms for all TTX treatments with values of 0.29 (90% CI: −0.26–0.83), −0.10 (90% CI: −0.80–0.61), and −0.66 (90% CI: −2.0–0.68) for 15 µg, 30 µg, and 45 µg TTX, respectively (Table 4). In addition, since the value of zero was contained within the upper and lower bounds of the 90% CI, all three TTX treatments were considered statistically similar to placebo. A scatter plot of the ΔΔQTcF—plasma TTX concentration relationship is shown in Figure 3. Linear regression was used to provide an estimated slope of −1.38 ms per ng mL^−1^ (90% CI: −3.27–0.51) with the value of zero contained within the bounds of the 90% CI.

### 2.8. Assay Sensitivity

To evaluate the sensitivity of the study in detecting QTc prolongation, 400 mg moxifloxacin was administered, and plasma moxifloxacin concentration was assessed up to 24 h post-dose. The largest change in baseline corrected QTcF between 400 mg moxifloxacin and placebo was seen at 4 h post-dose with a value of 14.9 ms (90% CI: 11.7–18.1 ms). Lower bound of the 90% CI of placebo and baseline corrected QTcF were > 5 ms at 11 of 12 post-dose time points, confirming assay sensitivity of the study. Assay sensitivity was also confirmed using the same C-QTc modeling with the *p*-value of the slope significant at *p* < 0.0001. The predicted maximum ΔΔQTcF at the geometric mean C_max_ of moxifloxacin exceeded 10 ms. For the 3 moxifloxacin active treatments (i.e., treatments E, G, and I) the lower bound of the 90% CI of maximum predicted ΔΔQTcF values exceeds 10 ms.

### 2.9. Heart Rate Effects

**Figure 4:**
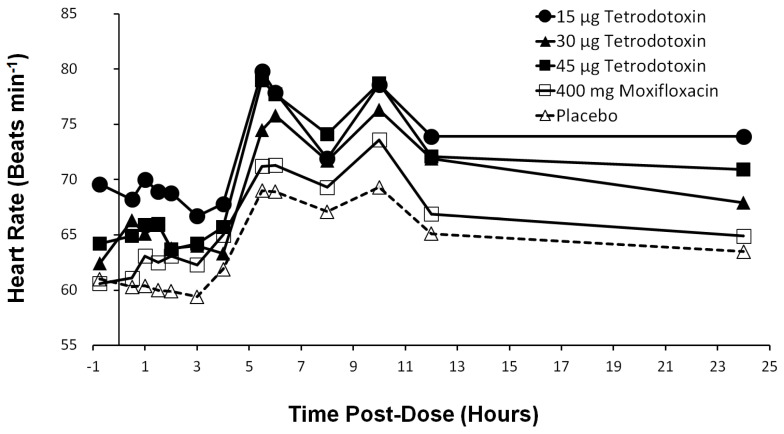
Heart rate in beats per minute across treatments (15 µg TTX (●), 30 µg TTX (▲), 45 µg TTX (■), 400 mg moxifloxacin (□), and placebo (∆)) at pre-dose and all post-dose time points.

The mean heart rate was ±1 bpm and similar to baseline from 0–4 h post-dose (Figure 4). At 4 h post-dose, the mean heart rate began to increase from baseline across all groups including placebo and peaked twice at 5.5 h and 10 h post-dose. This increased heart rate at both 5.5 h and 10 h post-dose occurred approximately 1.5 h after food consumption which is known to affect heart rate [29,30]. Heart rate began to decrease towards baseline after 10 h. 

### 2.10. PR and QRS Intervals

No trend in PR and QRS intervals was observed (Table S4 and Table S5, respectively). Mean changes from baseline values for PR and QRS intervals were minimal for all treatments and similar to placebo. No subject displayed a PR interval ≥ 220 ms and all QRS values were < 120 ms.

## 3. Discussion

TTX has long been used in Chinese and Japanese traditional medicine. Pufferfish extracts were used for centuries to alleviate neuralgia in patients affected by leprosy, reduce tetanus-related muscle spasms, and relieve pain resulting from rheumatoid arthritis [31,32]. Today, modern pharmacological studies have supported the beneficial effects of TTX on pain [15,17,33,34,35,36]. Despite the current need for effective, non-addictive analgesics, concern around toxicity and potential for cardiac arrythmias have limited wider clinical acceptance of TTX. This is the first published study addressing the safety, tolerability, and proarrhythmic liability of TTX at clinically relevant doses in healthy subjects. 

In this dose escalation study, therapeutic doses of 15 µg, 30 µg, and 45 µg TTX were observed to be safe and well tolerated. The majority of reported TEAEs were mild with no reported serious AEs, deaths, or AEs resulting in study withdrawal. The most common TEAEs (overall occurrence ≥ 5%) in TTX treatment groups were paresthesia, oral paresthesia, headache, dizziness, nausea, and myalgia. Safety assessments including vital signs, clinical laboratory tests, and 12-lead ECG showed no safety-relevant changes. Despite the role of TTX-sensitive VGSCs on the neuromuscular [11,23] or respiratory system functioning [24], the lack of clinically significant changes in motor control, muscle strength, detection of vibration, oxygen saturation, or peak expiratory flow rate indicate that the effect of low therapeutic concentrations of up to 45 µg TTX on these systems are not of clinical concern. Thus, the minimal adverse events profile and lack of changes in safety assessments performed in this study indicate therapeutic doses up to 45 µg TTX do not pose any safety concerns in healthy adults. Other in vitro and in vivo studies which directly evaluated cell lines, histological examinations of nerves and muscle tissues also support a lack of neurotoxic, myotoxic, and genotoxic activity with TTX [37,38,39,40].

The prominent role of VGSC on cardiac action potential is well established with immunohistochemical and electrophysiological studies demonstrating functional expression of the TTX-resistant cardiac VGSC (TTX-IC_50_ ≥ 1 µM) on mammalian cardiomyocytes [41]. However, the discovery of TTX-sensitive VGSC isoforms (TTX-IC_50_ ~10 nM) on human cardiomyocytes [25,26] has raised considerable debate on the proclivity of low nanomolar concentrations of TTX to induce myocardial dysfunction and arrhythmia in humans. This phase 1 study, performed in accordance to ICH E14 guidelines, presents the first assessment of the QTc liability of TTX in humans and demonstrates low nanomolar concentrations of TTX do not cause QTc interval prolongation of clinical or regulatory concern. The highest dose administered, 45 µg TTX corresponds to a plasma geometric C_max_ of 0.9914 ng mL^−1^ equivalent to approximately 3.11 nM TTX, thus confirming only TTX-sensitive VGSC isoforms were blocked by therapeutic doses of TTX in this study.

Single doses of 15 µg, 30 µg, and 45 µg TTX corresponding to plasma C_max_ dose increase of 3.3-fold, did not show any QT prolongation of the mean ΔΔQTcF interval at any time points investigated as determined from both mixed-effects model and C-QTc model. Upper limits of the two-sided 90% CIs were below the 10 ms threshold of regulatory concerns. Assay sensitivity was confirmed with moxifloxacin with values consistent with those reported for Avalox^®^ [42]. This study therefore establishes therapeutic TTX doses up to 45 µg are not expected to pose a cardiac repolarization liability of clinical or regulatory concern. No effects on PR and QRS intervals were observed. The increase in heart rate observed across all groups including placebo at 5.5 h and 10 h post-dose are likely due to food consumption and are in keeping with published data showing glycemic changes can influence heart rate with observed heart rate mean peak changes at about 1.5 h after food consumption [29,30]. There are some studies to suggest the QTc interval is impacted by gender [43,44] with females being more sensitive and susceptible to certain drugs that affect the I_Kr_ channel resulting in QT prolongation [45,46,47,48]. In this regard, no clinically relevant QT prolongation effect was observed despite the preponderance of females in TTX treatment arm (89% females).

Results from this study are consistent with findings from earlier preclinical and clinical observations during development of TTX. There was no significant inhibition of the human Ether-à-go-go-Related Gene (hERG) tail current between TTX and hERG channel in hERG transfected human embryonic kidney cells (HEK−293) exposed to 0.1 mg mL^−1^ of TTX for 15 min (WEX Pharmaceuticals Inc., data on file). Compounds that inhibit hERG current have been shown to prolong the cardiac action potential and hence QT interval in humans [49].

Lack of any cardiac liability with TTX exposure is further supported in reports of accidently intoxicated patients who were hospitalized after ingesting large quantities of TTX from improperly processed pufferfish. Patients who were placed on artificial ventilation to prevent hypoxemia showed no ECG abnormalities indicative of cardiac arrhythmias such as sinoatrial block, atrioventricular block, bundle branch block, or ventricular tachycardia/fibrillation [50]. Thus, cardiac conduction abnormalities observed in severely intoxicated patients (approximate blood TTX concentrations ≥ 40 µM) prior to admittance to the emergency department or intensive care units were ascribed to hypoxemia resulting from respiratory impairment rather than direct action of TTX on cardiomyocytes. Similar results were observed with ECG changes indicative of cardiac arrhythmias appearing only after complete respiratory arrest and subsequent drop in arterial oxygen in studies where guinea pigs [51] and dogs [52] were administered a lethal dose of TTX. 

Pharmacokinetic results obtained are consistent with findings from previous studies with healthy subjects (WEX Pharmaceuticals Inc., data on file). TTX was shown to have rapid absorption, reaching a peak plasma concentration within 1.5 h, and a half-life of approximately 4.5 h, irrespective of dose. Absorption and elimination of TTX were comparable across all TTX treatments as indicated by similar t_½_ and t_max_ values between treatments. Plasma TTX concentration decreased steadily following t_max_ and was undetectable (below the limit of quantification) in almost all subjects at 24 h post-dose. There were no relevant changes in steady-state C_max_ and AUC values which increased in proportion to dose. 

The principle limitation of this study was that the 45 ug TTX was the highest dose evaluated and thus, the results cannot be extrapolated to higher doses. Other limitations would typically be the small sample size and the use of C-QTc analysis which employs time-matched mean placebo correction in calculating ΔΔQTcF values rather than within-subject placebo treatment correction because subjects in C-QT studies do not cross-over to receive active or placebo treatment as is common in a traditional thorough-QT (TQT) study. A C-QT design was selected because studies have shown that triplicate ECGs recordings along with time-matched C-QTc samples in ascending dose trials provide data over a wide range of doses allowing for detection of a QT prolonging effect in small cohorts of subjects thereby enabling a study to minimize exposure of healthy individuals to unneeded medication at reduced cost compared to a traditional thorough-QT trial [53,54,55,56,57].

## 4. Conclusions

This study has confirmed that therapeutic doses of TTX has acceptable tolerability and PK profiles and does not pose toxicity concerns of clinical importance. The cardiac safety analysis demonstrated administration of TTX up to 45 µg is well tolerated and does not cause QTc interval prolongation of clinical or regulatory concern as determined from both the mixed-effects model and C-QTc analysis. The most frequently reported TEAE, accounting for 78% (7 out of 9 subjects in the treatment arm) of all TEAEs, was paresthesia and all were mild in severity. No serious or severe AEs and no withdrawals secondary to AEs occurred. 

## 5. Materials and Methods 

This study was conducted in accordance with Guidelines for Good Clinical Practice and the revised Declaration of Helsinki. All subjects provided a signed informed consent form prior to their participation in the study. All study documents were approved by Chesapeake International Review Board (Columbia, MD, USA) (Project Identification Code PRO00024161, 25 January 2018.) prior to study initiation. Study was performed on healthy adults confined at a single clinical site (Celerion Inc., Tempe, AZ, USA) between February 7 and March 24, 2018. Clinical trial registration with the National Institutes of Health (ClinicalTrial.gov Identifier: NCT04083833) was retrospective according to FDA Amendments Act of 2007. 

### 5.1. Study Population 

Study participants were healthy male and female subjects aged 18 to 55 years with a body mass index (BMI) between 18 and 32 kg m^−2^. To participate, subjects were required to have no clinically significant abnormal medical history, physical examination, neurological examination, vital signs, ECG, and clinical laboratory test results. Female subjects were of non-childbearing potential or using contraception. Subjects were excluded if they met any of the following conditions: resting heart rate <50 beats per minute (bpm) or >100 bpm; seated blood pressure <90/40 mmHg or >140/90 mmHg; sign or history of risk factors for Torsade de Pointes; prolonged QTcF interval >450 milliseconds (ms); sign or history of neurological or neuromuscular disease; history of hypersensitivity to fish; pregnant or breastfeeding.

### 5.2. Study Design

**Figure 5:**
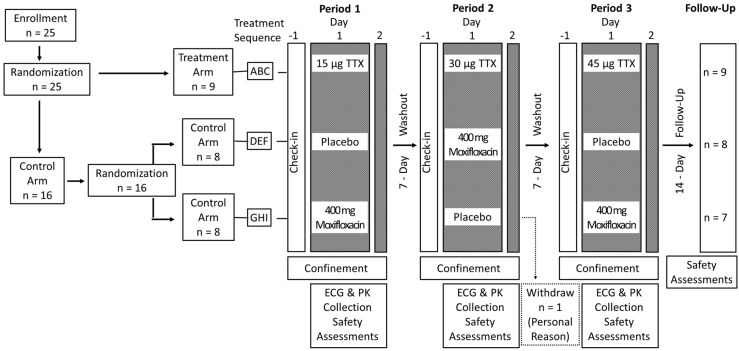
Schematic for this randomized, double-blind, placebo-, and positive-controlled, dose escalation study.

Study schematic is shown in Figure 5. A total of 25 eligible subjects were randomized in a double-blind manner to either a treatment or control arm. Subjects randomized to the treatment arm received single subcutaneous (s.c.) doses of 15 µg, 30 µg, and 45 µg TTX in sterile water (Halneuron^®^ manufactured by K.A.B.S. Laboratories Inc, Quebec, Canada for WEX Pharmaceuticals Inc.) over 3 periods with a 7-day washout interval between each period. Subjects assigned to the control arm using Celerion’s computerized randomization scheme were further randomized to one of two sequences (placebo-moxifloxacin-placebo or moxifloxacin-placebo-moxifloxacin) and received placebo and moxifloxacin in a crossover fashion over 3 periods. All subjects were admitted at least 12 h before dosing on day 1 of each period and remained domiciled for approximately 24 h until all assessments were completed on day 2. Moxifloxacin (400 mg, Bayer Pharmaceuticals, West Haven, CT, USA) was used as a positive control to demonstrate assay sensitivity. In crossover designed studies with healthy subjects, 400 mg moxifloxacin has been shown to prolong heart rate-corrected QT interval by 10–15 ms compared to placebo [42,58,59]. 

### 5.3. Randomization and Blinding 

All randomization was performed using Celerion’s computerized randomization scheme. The random allocation sequence was generated using the RANUNI function in SAS. The first 21 randomization numbers used a block size of three (seven blocks) for random assignment of one treatment and two controls within each block. The last block used a block size of four for random assignment of the treatment repeated twice and two controls. All data were stored in a password protected computer. All study personnel including participants, investigators, care providers, board-certified cardiologist, and all individuals assessing study outcomes were blinded during the study. A pharmacist not involved in the study was responsible for preparing the TTX, moxifloxacin, TTX-matched placebo, and moxifloxacin-matched placebo in a blinded manner.

### 5.4. Safety and Tolerability 

Safety and tolerability were evaluated on all subjects who received at least one dose of drug or placebo. Adverse events (AEs) were monitored throughout the study from day −1 to day 2 of each treatment period and at follow up 14 days after the end of study to ensure no further AEs had occurred. Verbatim term, preferred term using Medical Dictionary for Regulatory Activities (MedDRA) version 20.1 (MedDRA MSSO, McLean, VA, USA), system organ class (SOC), number and percentage of subjects reporting AEs in each treatment, severity, relationship to drug, and corrective action was reported for all AEs. 

Safety assessments included vital sign measurements (orthostatic blood pressure, heart rates, pulse oximetry), safety 12-lead electrocardiogram (ECG), clinical laboratory assessment (serum chemistry, hematology, urinalysis), peak expiratory flow (3 measurements per time point), with physical and neurological examinations (mental status, cranial nerves, deep tendon reflexes, cerebellar function by balance and coordination, gait, the sensory system by vibration and light touch or pinprick, the motor system using dynamometer for hand grip strength, leg raise against resistance assessment, and finger-nose coordination) were performed at screening. In addition, for each period: clinical laboratory assessment was repeated at day − 1; vital signs and safety ECGs were repeated within 24 h pre-dose, at 1 h post-dose, and at 24 h post-dose; pulse oximetry was monitored continuously for 4 h post-dose; neurological examination of motor system and sensory system by vibration and peak expiratory flow tests were repeated at 1 h and at 4.5 h post-dose. Concomitant medication was monitored throughout the study. AEs, vital signs, safety ECGs, hand grip strength, peak expiratory flow, and clinical laboratory data were presented using descriptive statistics. 

### 5.5. Pharmacokinetics

TTX and TTX-matched placebo along with moxifloxacin and moxifloxacin-matched placebo were administered following an overnight fast as single s.c. injections and single oral tablets, respectively, on day 1 of each treatment period. All subjects received both an injection and an oral tablet according to the randomization scheme. For example, subjects in the TTX treatment group received a TTX injection and a moxifloxacin-matched placebo tablet while subjects in the placebo group received an injection and an oral tablet of TTX-matched and moxifloxacin-matched placebos, respectively. Blood samples were collected for PK analysis following ECG extraction at the following time points: −0.75, −0.5, −0.25, 0.5, 1, 1.5, 2, 3, 4, 6, 8, 10, 12, and 24 h for each treatment period. An additional timepoint at 5.5 h was included for moxifloxacin and placebo groups to better characterize moxifloxacin’s t_max_ range and effect on QT interval since the maximum effect of moxifloxacin is often observed around 4 h post-dose. TTX and moxifloxacin plasma concentrations were measured at BRI Biopharmaceutical Research (Vancouver, BC, Canada) and Celerion Bioanalytical Laboratory (Lincoln, NE, USA), respectively, using validated liquid chromatography with tandem mass spectroscopy (LC-MS/MS) methods with 0.025 ng mL^−1^ and 25.0 ng mL^−1^ as lower limit of quantitation for TTX and moxifloxacin, respectively. In brief, aliquots of human plasma (K_2_EDTA) containing the analyte (moxifloxacin or TTX) and corresponding internal standard were extracted using a solid phase extraction procedure. The extracted samples were analyzed by an HPLC equipped with an AB SCIEX API 4000^™^ triple quadrupole mass spectrometer for moxifloxacin and a Waters Quattro^®^-Micro API with tandem quadrupole mass spectrometer for TTX, both using an electrospray ionization source. Positive ions were monitored in the multiple reaction monitoring (MRM) mode. Quantitation was determined using a weighted linear regression analysis (1/concentration^2^) of peak area ratios of the analyte and internal standard.

The following non-compartmental PK parameters were calculated from plasma TTX and moxifloxacin concentration-time data using Phoenix WinNonlin Version 7.0 software (Certara USA, Inc., Princeton, NJ, USA): C_max_, t_max_, t_½,_ AUC_0–t_ (area under concentration-time curve from time zero to time of last quantifiable concentration), and AUC_0–∞_ (area under concentration-time curve from time zero extrapolated to infinity). PK parameters were pooled for all subjects who received moxifloxacin while TTX PK parameters were summarized by TTX dose and were not calculated for subjects with less than 3 consecutive post-dose time points with quantifiable concentrations. Sample size (*n*), arithmetic mean, standard deviation (SD), coefficient of variance (CV%), standard error of the mean (SEM), minimum, median, maximum, geometric mean and geometric CV% were calculated for plasma TTX and moxifloxacin PK parameters. Dose proportionality of TTX PK parameters was evaluated using a regression approach [60]. A statistical linear relationship between natural log (ln)-transformed plasma PK parameters AUC_0–t_, AUC_0–∞_, and C_max_ for TTX and ln-transformed dose (taken as total daily dose) were fitted using a regression model with ln-transformed as a covariate.

### 5.6. Electrocardiography

Continuous 12-lead Holter monitoring (Global Instrumentation M12R Recorder, Manlius, NY, USA) was conducted for QT assessment from approximately 1-hour pre-dose to 24 h post-dose during each treatment period. Triplicate 10-second ECG recordings were extracted within a 5-min window from the continuous data at following time points relative to dosing (hours: −0.75, −0.5, −0.25, 0.5, 1, 1.5, 2, 3, 4, 5.5, 6, 8, 10, 12, 24) of each treatment period. Clinical staff ensured all subjects were awake during the ECG recordings to eliminate any autonomic QTc changes associated with sleep. ECG recordings were measured and classified by Antares software (AMPS LLC, New York, NY, USA). CAL ECG program (AMPS LLC, New York, NY, USA) was used for interval measurement on extracted ECG recordings and prespecified quality thresholds of ECG tracings were assessed by FAT-QT (AMPS LLC, New York, NY, USA). ECG recordings meeting safety and quality criteria thresholds were recorded in database. All ECG recordings not meeting specific safety and quality criteria thresholds and all waveforms identified for review by the automated algorithm were assigned to a single board-certified pharmaceutical industry experienced cardiologist (reader) at Celerion’s ECG core laboratory (Tempe, AZ, USA) for review. Cardiologist was blinded to subject, time, and treatment. A superimposed representative-complex method [61] was used for all interval analyses. QT interval, RR interval, heart rate, PR interval, and QRS duration were recorded. ECG waveform abnormalities including changes in T wave morphology and pathologic U waves were assessed and recorded in EGC data listings.

### 5.7. Data and Statistical Analysis

A sample size of 9 subjects for treatment arm and 16 subjects for control arm was deemed appropriate to provide > 80% power to detect a 1-sided 95% upper CI for ΔΔQTcF of 5 ms between TTX and placebo cohorts and to account for potential subject dropouts. The International Consortium for Innovation and Quality in Pharmaceutical Development and the Cardiac Safety Research Consortium (IQ-CSRC) study has shown that drug induced QTc prolongation can be detected in a small number of subjects using C-QTc modeling [54]. Additional studies [59,62] evaluating sample size in early phase single ascending dose studies have shown that 8 to 9 subjects per group will provide > 80% power to detect significant drug-induced QTc prolongation. Sample size in TTX treatment arm was also based on recent guidance by FDA that in a dose escalation study, 6 to 9 active subjects would provide an adequate set of observations at each dose level to evaluate a C-QTc relationship [55,63,64].

### 5.8. QT Analysis

Mean of triplicate ECG recordings was used as the value at each time point. Baseline values were derived from mean of 3 triplicate pre-dose QTc interval values (9 pre-dose measurements) measured on Day 1 of each corresponding period. Descriptive statistics including standard ECG parameters (heart rate, RR, PR, QRS, QT) plus baseline-adjusted Fridericia-corrected QTc (ΔQTcF) and baseline and placebo-adjusted QTcF (ΔΔQTcF) were examined. Since the treatment arm and control arm used different subjects, for the treatment arm, ΔΔQTcF was calculated as the difference in ΔQTcF (active treatment) minus time-matched placebo mean. Mean and 90% CI of ΔQTcF and ΔΔQTcF by time point and treatment were derived to determine if at any time point, the upper bounds of 2-sided 90% CI exceeded 10 ms. All subjects who had valid ECG data for post-dose time points during periods 1–3, were included in the primary analysis set. Within control arm, assay sensitivity was established if lower bound of the 90% CI of moxifloxacin ΔΔQTcF was greater than 5 ms for at least one post-dose time points.

Categorical analysis for each ECG parameter was performed at all time point in each period and summarized on frequency tables showing the count and percentage of subjects with ECG interval parameters exceeding the following threshold criteria: absolute QT and QTcF values >500 ms, >480 and ≤500 ms, >450 and ≤480 ms, or ≤450 ms and increases from baseline of ≥60 ms, ≥30 and <60 ms, or <30 ms. Descriptive statistics of heart rate, PR, and QRS intervals and changes in these parameters from baseline were also included.

For concentration-QTc relationship analysis, hysteresis or time delay between measured concentration and effect on QTcF, was first examined by plotting mean TTX concentration and mean ΔΔQTc values over time for each dose level and further evaluated with loop plots. Since an absence of hysteresis was observed (data not shown), the relationship between QTc and plasma TTX concentration was assessed using a linear mixed effect model with ΔQTcF as the dependent variable, time as the fixed effect, baseline and TTX plasma concentration as continuous covariates, and subject as the random effect (TTX model) with an assumption of unstructured covariance matrix. A similar model using moxifloxacin plasma concentration was performed to determine assay sensitivity but with subject nested in sequence (since the control arm had 2 sequences) as the random effect. For the TTX model, four pre-specified models were assessed: a model with random intercept; a model with random intercept with quadratic effect on concentration; a model without random effects on slope or intercept; and a model without random effects on slope or intercept with quadratic effect on concentration. Diagnostic plots using studentized residual and QQ plot were used to evaluate the adequacy of each model fit to the assumption of normality and impact on quantifying the concentration-response relationship [65]. The model with the lowest Akaike Information Criterion Corrected (AICC) value was selected. After establishing linearity, a linear regression of ΔΔQTcF versus time-matched plasma TTX concentrations was performed to predict both the individual and population average ΔΔQTcF (at geometric mean maximum plasma concentration for population average) and their corresponding two-sided 90% CI for each of 15 µg, 30 µg, and 45 µg TTX doses.

## Figures and Tables

**Table 1:** Demographic and baseline characteristics of subjects in study.

Parameter	Treatment Arm	Control Arm		All Subjects
	ABC	DEF	GHI	Total
	*n* = 9	*n* = 8	*n* = 8	*n* = 25
Female, *n* (%)	8 (89%)	5 (63%)	6 (75%)	19 (76%)
Male, *n* (%)	1 (11%)	3 (38%)	2 (25%)	6 (24%)
Race, *n* (%) Black/African American	0 (0%)	1 (13%)	1 (13%)	2 (8%)
Caucasian	9 (100%)	7 (88%)	7 (88%)	23 (92%)
Ethnicity, *n* (%) Hispanic/Latino	8 (89%)	5 (63%)	6 (75%)	19 (76%)
Not Hispanic/Latino	1 (11%)	3 (38%)	2 (25%)	6 (24%)
Age (years) ^a^ Mean ± SD (Range)	38.7 ± 9.90 (24–53)	36.6 ± 9.61 (24–49)	34.5 ± 11.39 (18–46)	36.7 ± 10.02 (18–53)
Weight (kg) Mean ± SD (Range)	76.1 ± 13.86 (54.0–104.6)	77.3 ± 14.65 (53.9–98.7)	76.2 ± 13.21 (51.2–96.9)	76.5 ± 13.34 (51.2–104.6)
Height (cm) Mean ± SD (Range)	160.7 ± 9.63 (145–181)	165.1 ± 11.76 (156–184)	161.0 ± 7.35 (153–174)	162.2 ± 9.55 (145–184)
BMI (kg m^−2^) ^b^ Mean ± SD (Range)	29.24 ± 2.277 (25.68–31.97)	28.19 ± 3.326 (22.03–31.77)	29.22 ± 3.532 (21.38–31.93)	28.90 ± 2.973 (21.38–31.97)

^a^ Age is calculated from birth to date of first dosing; ^b^ BMI is the abbreviation for body mass index.

**Table 2:** Treatment emergent adverse events.

Adverse Event	15 µg TTX ^a^ (%) (Severity/Relationship)		30 µg TTX (%) (Severity/Relationship)		45 µg TTX (%) (Severity/Relationship)		Overall TTX (%)	400 mg ^b^ Moxifloxacin (%)	Placebo ^c^ (%)
Total TEAEs ^d^	9 (100%)		24 (100%)		46 (100%)		79 (100%)	14 (100%)	8 (100%)
Arthralgia	0		0		1 (2%)		1 (1%)	0	0
					Mild	Possibly			
Back Pain	0		1 (4%)		1 (2%)		2 (3%)	0	0
			Mild	Unlikely	Moderate	Unlikely			
Chest Discomfort	0		0		1 (2%)		1 (1%)	0	0
					Mild	Possibly			
Chills	0		0		1 (2%)		1 (1%)	1 (7%)	0
					Mild	Possibly			
Cough	0		1 (4%)		0		1 (1%)	0	0
			Mild	Unlikely					
Dizziness	1 (11%)		3 (13%)		4 (9%)		8 (10%)	2 (14%)	1 (13%)
	Mild	Probably	3x Mild	3x Probably	4x Mild	4x Probably			
Dry Throat	0		0		1 (2%)		1 (1%)	0	0
					Mild	Probably			
Ear Pruritus	0		1 (4%)		0		1 (1%)	0	0
			Mild	Possibly					
Fatigue	0		1 (4%)		0		1 (1%)	0	0
			Mild	Unlikely					
Feeling Hot	0		0		1 (2%)		1 (1%)	0	1 (13%)
					Mild	Possibly			
Headache	3 (33%)		2 (8%)		4 (9%)		9 (11%)	1 (7%)	2 (25%)
	2x Mild 1x Moderate	2x Probably1x Possibly	1x Mild 1x Moderate	1x Probably 1x Possibly	2x Mild 2x Moderate	4x Probably			
Hyperhidrosis	1 (11%)		0		0		1 (1%)	0	0
	Mild	Possibly							
Myalgia	1 (11%)		0		3 (7%)		4 (5%)	2 (14%)	1 (13%)
	Mild	Possibly			3x Mild	1x Probably 2x Unlikely			
Nausea	0		2 (8%)		2 (4%)		4 (5%)	1 (7%)	0
			2x Mild	2x Probably	2x Mild	2x Probably			
Papule	0		2 (8%)		0		2 (3%)	0	0
			2x Mild	2x Possibly					
Paresthesia	2 (22%)		6 (25%)		10 (22%)		18 (23%)	0	0
	2x Mild	2x Probably	6x Mild	6x Probably	10x Mild	9x Probably 1x Possibly			
Paresthesia Oral	0		3 (13%)		8 (17%)		11 (14%)	0	0
			3x Mild	3x Probably	8x Mild	8x Probably			
Pharyngeal Paresthesia	0		0		3 (7%)		3 (4%)	0	0
					3x Mild	3x Probably			
Productive Cough	0		0		1 (2%)		1 (1%)	0	0
					Mild	Possibly			
Pruritus	0		1 (4%)		2 (4%)		3 (4%)	0	0
			Mild	Possibly	2x Mild	2x Possibly			
Rash Erythematous	0		0		1 (2%)		1 (1%)	0	0
					Mild	Possibly			
Tachycardia	1 (11%)		0		0		1 (1%)	0	0
	Mild	Possibly							
Throat Tightness	0		0		1 (2%)		1 (1%)	0	0
					Mild	Probably			
Vessel Puncture Site Pain	0		1 (4%)		0		1 (1%)	1 (7%)	0
			Mild	Unrelated					
Wheezing	0		0		1 (2%)		1 (1%)	0	0
					Mild	Possibly			

^a^ TTX is abbreviation for tetrodotoxin; ^b, c^ For each TTX-related AE, the corresponding results for moxifloxacin and placebo groups were included for comparison; ^d^ TEAE is abbreviation for treatment emergent adverse events.

**Table 3:** Plasma tetrodotoxin pharmacokinetics parameters.

Parameters	15 µg TTX ^a^ (*n* = 9)	30 µg TTX (*n* = 9)	45 µg TTX (*n* = 9)
**AUC_0–∞_ (h × ng mL^−1^)**			
**Geometric Mean**	1.9612	4.0094	6.4983
**Geometric (CV%)**	(31.8)	(17.6)	(10.0)
**C_max_ (ng mL^−1^)**			
**Geometric Mean**	0.3046	0.5807	0.9914
**Geometric (CV%)**	(30.3)	(17.4)	(16.2)
**T_max_ (h)**			
**Median**	1.50	1.50	1.50
**(Min, Max)**	(1.00, 2.00)	(1.00, 1.51)	(1.00, 2.00)
**t_½_ (h)**			
**Arithmetic Mean**	4.62	4.54	4.28
**Standard Deviation**	(±1.84)	(±0.39)	(±1.35)

^a^ TTX is abbreviation for tetrodotoxin.

**Table 4:** Slope and intercept from the exposure-response analysis of ΔΔQTcF by tetrodotoxin treatment.

Treatment	Geometric Mean C_max_ (ng mL^−1^)	Predicted ΔΔQTcF (ms) (90% CI)	Model Slope (ms (ng mL^−1^)^−1^) (90% CI)	Model Intercept (ms) (90% CI)
**15 µg TTX ^a^**	0.3046	0.285 (−0.255, 0.825)	−1.378 (−3.266, 0.510)	−0.705 (−0.131, 1.540)
**30 µg TTX**	0.5807	−0.096 (−0.798, 0.607)		
**45 µg TTX**	0.9914	−0.661 (−2.003, 0.680)		

^a^ TTX is abbreviation for tetrodotoxin.

## Supplementary Materials

The following are available online at https://www.mdpi.com/2072-6651/12/8/511/s1, Table S1: Blood pressure measurements, Table S2: Hand grip strength assessment, Table S3: Peak expiratory flow performance, Table S4: PR interval measurements, Table S5: QRS interval measurements.

## Abbreviations

The following abbreviations are used in this manuscript:

AE	adverse event
AUC	area under the curve
bpm	beats per minute
C_max_	maximum plasma concentration
CI	confidence interval
C-QTc	corrected concentration-QT interval
CV%	coefficient of variance
hERG	Human Ether-à-go-go-Related Gene
ICH E14	International Conference on Harmonization E14 guidance
PK	pharmacokinetics
QTcF	Fridericia corrected QT interval
ΔQTcF	Baseline adjusted Fridericia-corrected QTc
ΔΔQTcF	Baseline and placebo adjusted Fridericia-corrected QTc
TEAE	treatment emergent adverse event
t_max_	time at C_max_
TTX	tetrodotoxin
VGSC	voltage-gated sodium channel
