# Supplementary material for: Safety, Tolerability, Pharmacokinetics, and Concentration-QTc Analysis of Tetrodotoxin: A Randomized, Dose Escalation Study in Healthy Adults
Source: Toxins (Basel). 2020 Aug 9;12(8):511. doi: 10.3390/toxins12080511 (PMC7472037; doi:10.3390/toxins12080511)
Supplement: Supplementary file 1 [file toxins-12-00511-s001.pdf]

Supplementary Materials: Safety, Tolerability, Pharmacokinetics, and Concentration-QTc Analysis of Tetrodotoxin: A Randomized, Dose Escalation Study in Healthy Adults

Mojgan Kavoosi, Terry E. O'Reilly, Mehran Kavoosi, Peng Chai, Caroline Engel, Walter Korz, Christopher C. Gallen and Robert M. Lester

Table S1. Blood pressure measurements.

Time	Blood Pressure	15 µg TTX[a] (mmHg)	30 µg TTX (mmHg)	45 µg TTX (mmHg)	400 mg Moxifloxacin (mmHg)	Placebo (mmHg)
Screening[b]	Systolic		119 ± 8.57		118 ± 10.99	
	Diastolic		79.3 ± 1.33		78.3 ± 6.89	
Baseline	Systolic	114 ± 8.54	112 ± 8.33	112 ± 9.65	116 ± 8.28	116 ± 11.41
	Diastolic	74.3 ± 4.95	74.6 ± 7.09	72.4 ± 7.37	75.7 ± 5.79	75.3 ± 6.57
1 Hour Post-dose	Systolic	112 ± 14.2	115 ± 10.5	120 ± 11.1	116 ± 12.1	117 ± 14.6
	Diastolic	78.1 ± 8.01	78.4 ± 8.2	78.3 ± 5.52	77.3 ± 8.48	76.8 ± 7.51
24 Hours Post-dose	Systolic	–	–	111 ± 8.7	114 ± 8.5	115 ± 18.2
	Diastolic	–	–	75.9 ± 6.33	75.8 ± 6.88	73.4 ± 9.13

[a] TTX is abbreviation for tetrodotoxin. [b] Values are for treatment arm (15 µg TTX, 30 µg TTX and 45 µg TTX) and control arm (moxifloxacin and placebo).

Table S2. Hand grip strength assessment

Time	Hand Location	15 µg TTX[a] (kg)	30 µg TTX (kg)	45 µg TTX (kg)	400 mg Moxifloxacin (kg)	Placebo (kg)
Screening[b]	Right		32.1 ± 10.5		37.7 ± 9.4	
	Left		31.0 ± 10.5		34.4 ± 9.1	
1 Hour Post-dose	Right	28.6 ± 10.0	27.5 ± 8.7	28.4 ± 8.1	34.7 ± 10.7	35.9 ± 11.3
	Left	28.1 ± 9.1	26.5 ± 9.0	28.6 ± 9.5	32.3 ± 10.5	32.6 ± 10.4
4.5 Hours Post-dose	Right	29.8 ± 10.6	28.5 ± 9.2	27.2 ± 7.8	35.8 ± 11.1	36.1 ± 10.0
	Left	28.8 ± 8.1	28.0 ± 7.1	26.9 ± 7.0	32.7 ± 9.3	34.3 ± 12.1

[a] TTX is abbreviation for tetrodotoxin. [b] Values are for treatment arm (15 µg TTX, 30 µg TTX and 45 µg TTX) and control arm (moxifloxacin and placebo).

Table S3. Peak expiratory flow performance.

Time	15 µg TTX[a] (L/min)	30 µg TTX (L/min)	45 µg TTX (L/min)	400 mg Moxifloxacin (L/min)	Placebo (L/min)
Screening[b]		437.0 ± 92.3		489.4 ± 95.7	
1 Hour Post-dose	415.9 ± 75.3	424.1 ± 118.5	410.3 ± 121.9	464.0 ± 93.2	482.5 ± 87.6
4.5 Hours Post-dose	422.8 ± 97.4	435.6 ± 119.2	407.4 ± 124.5	464.7 ± 83.5	484.9 ± 89.7

[a] TTX is abbreviation for tetrodotoxin. [b] Values are for treatment arm (15 µg TTX, 30 µg TTX and 45 µg TTX) and control arm (moxifloxacin and placebo).

Table S4. PR interval measurements.

Time	15 µg TTX[a] (ms)	30 µg TTX (ms)	45 µg TTX (ms)	400 mg Moxifloxacin (ms)	Placebo (ms)
**Baseline**	159.9 ± 15.1	161.2 ± 19.9	160.4 ± 23.2	162.8 ± 23.4	165.3 ± 23.3
**0.5 Hour**	160.1 ± 14.9	162.4 ± 20.3	163.3 ± 22.9	160.6 ± 24.7	164.3 ± 24.3
**1 Hour**	159.6 ± 19.4	163.7 ± 19.8	163.1 ± 23.4	160.9 ± 24.8	164.4 ± 22.9
**1.5 Hour**	154.3 ± 17.4	163.3 ± 22.9	161.2 ± 23.8	161.1 ± 24.0	163.8 ± 25.6
**2 Hour**	157.6 ± 17.2	163.1 ± 19.3	158.1 ± 22.6	161.5 ± 23.2	164.1 ± 23.5
**3 Hour**	157.1 ± 16.4	160.8 ± 20.8	154.8 ± 23.4	162.0 ± 22.2	162.4 ± 24.7
**4 Hour**	160.6 ± 18.3	156.3 ± 23.8	157.3 ± 24.0	160.5 ± 24.2	162.6 ± 23.5
**5.5 Hour**	154.4 ± 20.2	160.1 ± 16.0	154.0 ± 20.3	159.1 ± 18.3	164.8 ± 19.2
**6 Hour**	152.7 ± 16.5	154.0 ± 16.3	152.9 ± 21.6	160.9 ± 18.2	163.6 ± 19.4
**8 Hour**	157.7 ± 22.1	156.9 ± 22.0	154.4 ± 21.8	158.6 ± 18.7	163.1 ± 20.6
**10 Hour**	152.9 ± 16.4	153.9 ± 20.0	152.1 ± 19.4	157.2 ± 17.1	160.1 ± 18.7
**12 Hour**	152.0 ± 17.1	157.3 ± 22.2	156.9 ± 23.2	159.4 ± 20.6	161.9 ± 22.2
**24 Hour**	157.9 ± 19.1	161.2 ± 21.6	159.4 ± 24.3	161.4 ± 24.7	162.3 ± 24.0

[a] TTX is abbreviation for tetrodotoxin.

Table S5. QRS interval measurements.

Time	15 µg TTX[a] (ms)	30 µg TTX (ms)	45 µg TTX (ms)	400 mg Moxifloxacin (ms)	Placebo (ms)
**Baseline**	81.7 ± 8.5	81.9 ± 8.7	82.0 ± 9.6	80.4 ± 6.7	79.4 ± 5.9
**0.5 Hour**	81.1 ± 8.8	81.7 ± 8.2	81.7 ± 8.8	79.7 ± 5.9	79.9 ± 6.1
**1 Hour**	81.7 ± 8.9	82.0 ± 9.2	82.2 ± 8.5	81.1 ± 6.6	79.9 ± 5.6
**1.5 Hour**	82.7 ± 9.1	81.7 ± 8.8	83.2 ± 11.8	82.0 ± 7.5	79.9 ± 5.8
**2 Hour**	81.2 ± 8.4	81.1 ± 8.9	83.4 ± 12.3	80.2 ± 5.5	79.1 ± 5.6
**3 Hour**	82.3 ± 8.4	81.3 ± 8.8	84.3 ± 11.0	80.0 ± 5.8	80.6 ± 6.2
**4 Hour**	81.0 ± 8.6	81.1 ± 8.5	82.7 ± 9.2	80.8 ± 6.5	79.8 ± 6.1
**5.5 Hour**	82.8 ± 10.4	83.6 ± 10.2	83.3 ± 9.1	83.3 ± 7.2	80.7 ± 6.0
**6 Hour**	83.3 ± 9.0	82.0 ± 7.9	83.3 ± 10.0	81.3 ± 5.8	79.9 ± 6.1
**8 Hour**	81.1 ± 9.5	79.8 ± 8.8	82.2 ± 10.0	79.6 ± 5.4	78.6 ± 5.8
**10 Hour**	82.0 ± 9.8	83.4 ± 8.8	82.7 ± 9.5	80.2 ± 6.1	80.3 ± 6.2
**12 Hour**	81.3 ± 8.8	81.6 ± 10.0	82.7 ± 11.0	79.9 ± 6.1	79.2 ± 6.2
**24 Hour**	80.6 ± 8.5	81.6 ± 7.8	80.8 ± 8.8	80.0 ± 5.9	79.8 ± 6.1

[a] TTX is abbreviation for tetrodotoxin.
